# Differential regulation of the water channel protein aquaporins in chondrocytes of human knee articular cartilage by aging

**DOI:** 10.1038/s41598-021-99885-7

**Published:** 2021-10-14

**Authors:** Bong Soo Kyung, Koo Whang Jung, Woo Jin Yeo, Hye Kyung Seo, Yong-Soo Lee, Dong Won Suh

**Affiliations:** Research Center for Cartilage Regeneration, Barunsesang Hospital, #75-5, Yatap-ro, Seongnam-si, 13497 Gyeonggi-do Republic of Korea

**Keywords:** Molecular biology, Medical research

## Abstract

Knee cartilage is in an aqueous environment filled with synovial fluid consisting of water, various nutrients, and ions to maintain chondrocyte homeostasis. Aquaporins (AQPs) are water channel proteins that play an important role in water exchange in cells, and AQP1, -3, and -4 are known to be expressed predominantly in cartilage. We evaluated the changes in AQP expression in chondrocytes from human knee articular cartilage in patients of different ages and identified the key factor(s) that mediate age-induced alteration in AQP expression. The mRNA and protein expression of AQP1, -3 and -4 were significantly decreased in fibrocartilage compared to hyaline cartilage and in articular cartilage from older osteoarthritis patients compared to that from young patients. Gene and protein expression of AQP1, -3 and -4 were altered during the chondrogenic differentiation of C3H10T1/2 cells. The causative factors for age-associated decrease in AQP included H_2_O_2_, TNFα, and HMGB1 for AQP1, -3, and -4, respectively. In particular, the protective effect of AQP4 reduction following HMGB1 neutralization was noteworthy. The identification of other potent molecules that regulate AQP expression represents a promising therapeutic approach to suppress cartilage degeneration during aging.

## Introduction

Osteoarthritis (OA) is the most prevalent joint disorder characterized by the progressive destruction of the cartilage extracellular matrix (ECM), subchondral bone thickening, and osteophyte formation, and is a major cause of disability and reduced quality of life in older adults^1^. Articular cartilage tissue has poor repair capacity due to the lack of blood vessels and a paucity of resident progenitor stem cells^2^. Therefore, most treatments for cartilage disorders, including OA, are dependent on pain relief or surgery. Increase in the onset of articular cartilage diseases due to an increase in population with OA risk factors including age, obesity, and sports injuries has stimulated research for the development of regenerative medicine strategies such as platelet-rich plasma (PRP) treatment or stem cell therapy to repair or regenerate cartilage^3–6^.

Articular cartilage ECM plays an important role in mediating chondrocyte homeostasis and metabolism, as well as cellular structure, and is predominantly composed of proteoglycans (mainly aggrecan), hyaluronic acid, collagen fibers (mainly type II collagen), and the cell adhesion molecule integrin^7^. An imbalance between anabolic synthesis and catabolic degradation of the major matrix components such as proteoglycans and collagen II is the primary cause of cartilage degeneration^8,9^. Cartilage damage is caused by a variety of factors, including physical trauma, inflammation, free radicals, and aging, and there are still several mechanisms that contribute to the pathogenesis of cartilage disorders that remain to be elucidated^10–12^. Recently, there has been a growing interest in cartilage degeneration due to aging because older adults are very susceptible to all of the causes of cartilage disorders, including alterations in the innate immune system caused by infection or injury^13^. Aging leads to various changes in chondrocytes and ECM of articular cartilage characterized by cellular senescence, proteolysis, advanced glycation, calcification, and even tissue destruction^14^. Ultimately, these intra- and extracellular changes are driven by the dysfunction of cellular homeostasis.

Knee cartilage is in an aqueous environment filled with synovial fluid consisting of water, various nutrients, and ions to maintain chondrocyte homeostasis as well as the discharge of waste products from chondrocytes^15^. Articular cartilage itself is also a highly hydrated tissue containing 65%–80% water, and fluid exchange via the ECM is vital for supplying various nutrients such as oxygen and glucose to chondrocytes^16^. Therefore, understanding the function of the molecules mediating between the aqueous environment and chondrocytes is crucial because the dysfunction of these molecules could directly lead to defects in cartilage functions.

Aquaporins (AQPs) are water channel proteins that play an important role in water exchange in cells, although some AQPs can carry small uncharged solutes, such as glycerol, carbon dioxide, ammonia, and urea^17^. The AQPs are responsible for a variety of biological processes involving movement of water including brain function, cell migration, transepithelial fluid transport, skin hydration, and even inflammatory response^18,19^. Therefore, certain defects in AQP function have been implicated in the pathophysiology of cells or tissues^20,21^. To date, 13 AQPs have been identified, of which AQP1, -3, and -4 are known to be expressed predominantly in cartilage^22–27^. In particular, AQP1 and AQP3 are involved in metabolic water regulation in the articular cartilage of load-bearing joints, demonstrating that AQPs play an important role in maintaining the basic morphology of cartilage tissue as well as the water or ionic homeostasis in the cartilage^22,24^.

The exact molecular mechanism of senescence of chondrocytes has not been fully elucidated. Moreover, the pivotal molecule that is sensitive to water or ion transport and osmotic changes in the extracellular environment and regulates AQP expression in articular cartilage remains to be identified. We hypothesized that, among the various detrimental factors, aging may induce certain alterations in AQP expression in knee articular cartilage, and suggest that control of the potent molecules that regulate AQP expression would be a promising therapeutic strategy for age-associated cartilage destruction caused by a defect in AQP.

## Results

### Decreased expression of AQPs in fibrocartilage and aged cartilage from osteoarthritis patients

**Figure 1:**
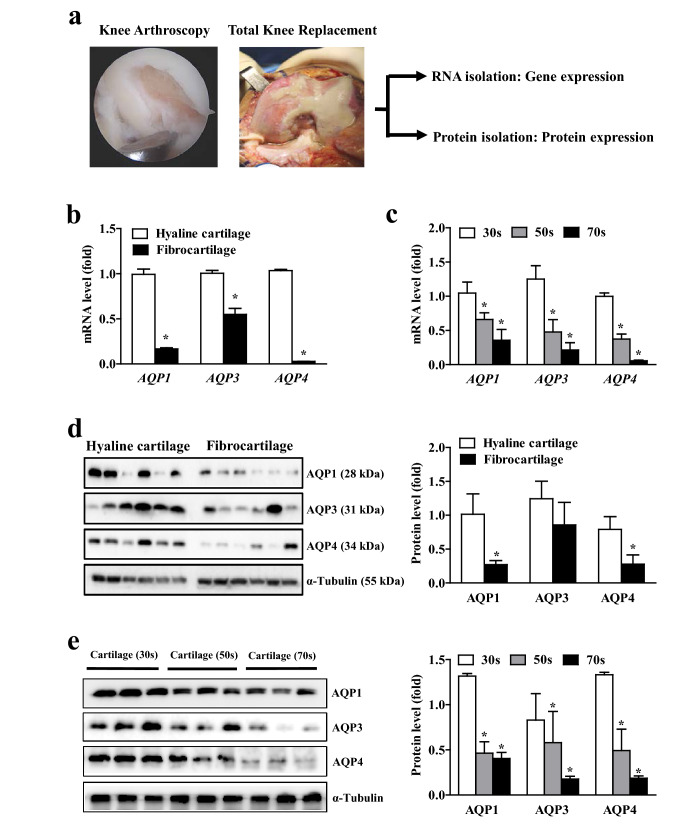
Aquaporin expressions in fibrocartilage and aged cartilage from osteoarthritis patients. (**a**) knee arthroscopy (left) and knee joint images during total knee replacement (right). Picture is a representative of independent image. Knee articular cartilage, the white or yellowish tissue that covers the ends of bones was harvested during arthroscopic surgery or total knee replacement, and used for total RNA and protein extraction, respectively. (**b**) Expressions of aquaporin (AQP)1, -3 and -4 mRNA between isolated hyaline cartilage and fibrocartilage (n = 12). Hyaline cartilage and fibrocartilage is defined as a normal intact cartilage apart from damaged area and a cartilage recovered from traumatic injury or degenerative tears over time, respectively. (**c**) Expression of AQP1, -3 and -4 mRNA in different aged cartilage tissues. All the cartilage tissues of 30 s (n = 12) and 50 s-aged (n = 14) patients were obtained by knee arthroscopy, and 70 s-aged (n = 14) cartilage tissues were acquired during total knee replacement. (**d**) Western blot analyses for AQP1, -3 and -4 protein expression between intact hyaline cartilage and fibrocartilage (n = 6 per group). Densitometric analyses of the western blots are shown on the right. (**e**) AQP1, -3 and -4 protein expression in different aged cartilage tissues (n = 3 per group). Densitometric analyses of the western blots are shown on the right. Data represent means ± standard errors of the means. *P < 0.05.

To determine whether AQPs are associated with cartilage physiology, we initially analyzed the expression of AQPs in cartilage tissues isolated from patients who were surgically treated for osteoarthritis of the knee by arthroscopic surgery or total knee replacement surgery (Fig. 1a). Compared to intact hyaline cartilage, fibrocartilage that had been injured previously showed substantial reduction in AQP1, -3 and -4 expression both at the mRNA and protein levels (Fig. 1b,d). As aging is one of the most deleterious factors for cartilage degenerations, we examined the expression of AQPs in cartilage tissues from different age groups. As shown in Fig. 1c,e, the mRNA and protein levels of all three types of AQPs were markedly decreased during aging. These results indicate that AQP1, -3 and -4 are potent markers of injury- and age-induced cartilage degeneration.

### Expression of AQPs during chondrogenic differentiation of C3H10T1/2 mesenchymal stem cell

**Figure 2:**
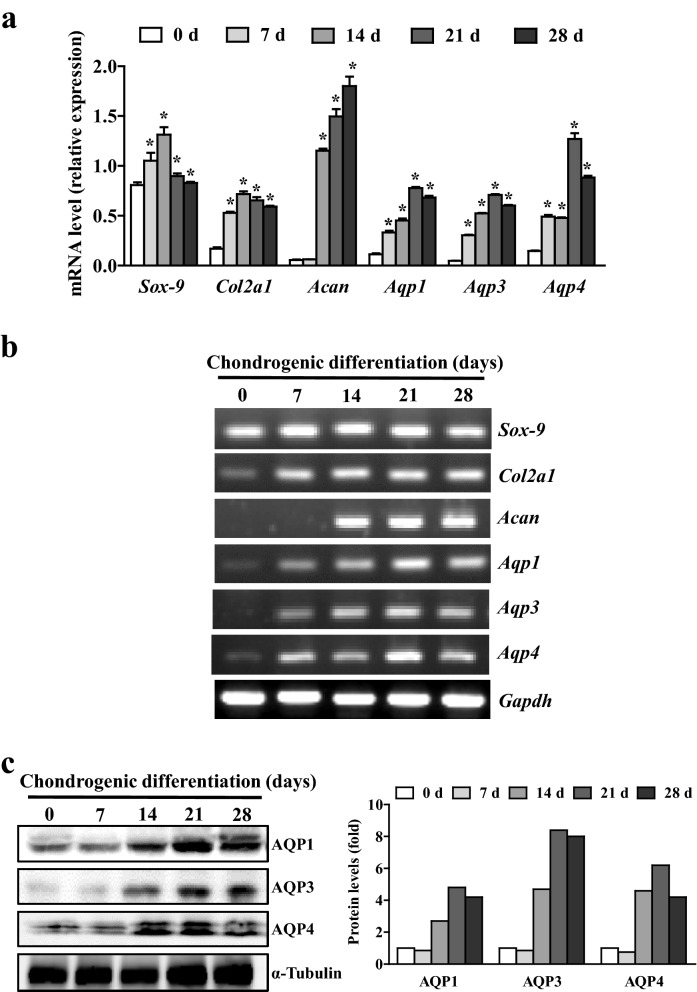
Aquaporin expressions during chondrogenic differentiation of C3H10T1/2 mesenchymal stem cell. (**a**) Expression of AQP1, -3 and -4 mRNA as well as chondrogenic marker genes in C3H10T1/2 mesenchymal stem cell treated with 100 ng of BMP2 for the indicated time periods (n = 4 per each designated time). (**b**) Agarose gel electrophoresis pictures of the PCR products from the transcript analyses of each RT-qPCR result in panel ‘(**a**)’. (**c**) Western blot analyses for AQP1, -3 and -4 protein expression during chondrogenic differentiation of C3H10T1/2 cells. Densitometric analyses of the western blots are shown on the right.

To examine whether AQPs play an important role in cartilage physiology, we examined the correlation between AQP expression and chondrocyte differentiation using C3H10T1/2 cells, a well-known in vitro model of chondrogenesis in the presence of BMP2^28^. As shown in Fig. 2a, the mRNA expression of the chondrogenic markers, SRY-box transcription factor 9 (Sox-9), collagen type II alpha 1 chain (Col2a1), and aggrecan (Acan) were markedly increased during chondrogenic differentiation in C3H10T1/2 cells. Interestingly, expression of all three types of AQP genes were also upregulated during the same differentiation period. The amplified PCR products for the all the genes that were evaluated revealed a clear correlation between the expression of chondrogenic genes and AQPs (Fig. 2b). AQP protein expression was also upregulated in C3H10T1/2 cells, with the highest levels at 21 days after chondrogenic differentiation, in accordance with mRNA expression (Fig. 2c). Taken together, these results suggest that AQPs may be involved in chondrocyte maturation and physiological functions of knee articular cartilage.

### Changes in AQP expression by aging-associated factors, reactive oxygen species (ROS) and pro-inflammatory cytokines

**Figure 3:**
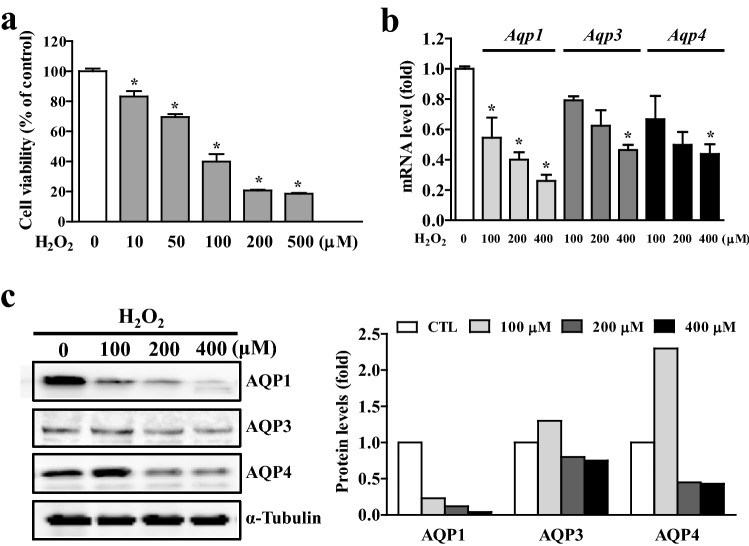
Aquaporin expression levels in chondrocytes by aging-associated factors, ROS. (**a**) Cell viability of chondrogenic C3H10T1/2 cells with a different dose of H_2_O_2_. (**b**) Expressions of AQP1, -3 and -4 mRNA in the presence of H_2_O_2_. (**c**) Western blot analyses for AQP1, -3 and -4 protein expression by a different dose of H_2_O_2_. Densitometric analyses of the western blots are shown on the right.

Aging is a well-known risk factor for the development of osteoarthritis (OA)^29^, and our data revealed that mRNA and protein levels of all three types of AQP were markedly decreased with aging (Fig. 1c,e). To examine whether chondrocyte AQP expression is directly regulated by age-associated factors, we treated differentiated C3H10T1/2 cells with hydrogen peroxide (H_2_O_2_), a major ROS. To confirm the H_2_O_2_ responsiveness of the chondrogenic cells, we initially examined cell viability, which gradually decreased following the ROS challenge, showing higher toxicity at 200–500 μM (Fig. 3a). As shown in Fig. 3b, mRNA levels of AQP1, -3, and -4 were significantly decreased by H_2_O_2_ administration. Interestingly, AQP1 showed higher sensitivity to H_2_O_2_ than to AQP3 and AQP4. Western blot analysis revealed that the protein levels of all three types of AQP were also markedly reduced by H_2_O_2_ at higher concentrations (Fig. 3c). These data suggest that chondrocyte-AQPs are affected by age-associated detrimental factors such as ROS, and H_2_O_2_.

**Figure 4:**
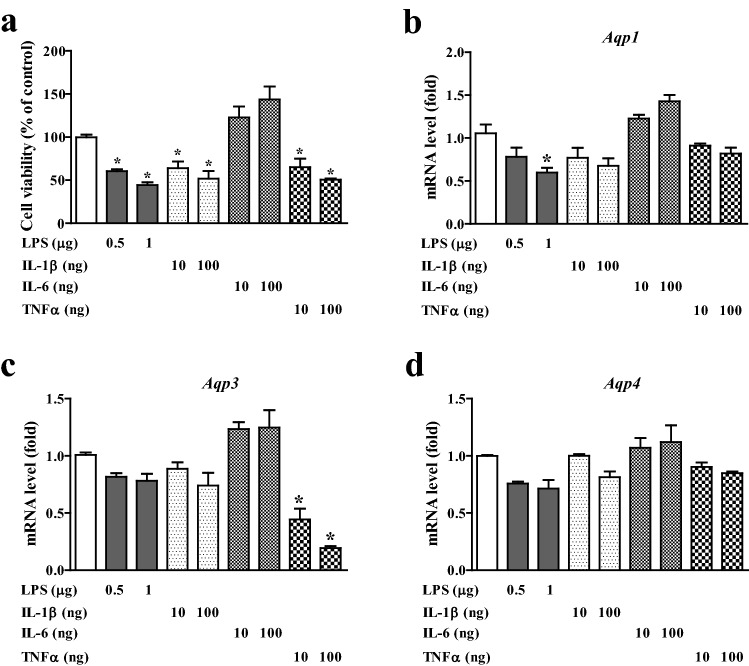
Aquaporin expression levels in chondrocytes by inflammatory cytokines. (**a**) Cell viability of chondrogenic C3H10T1/2 cells by pro-inflammatory molecules, lipopolysaccharides (LPS), interleukin 1 beta (IL-1β), IL-6 and tumor necrosis factor alpha (TNF-α). (**b**–**d**) mRNA expressions of AQP1 (**b**), -3 (**c**) and -4 (**d**) by LPS, IL-1β, IL-6 and TNF-α.

Inflammatory cytokines are critical mediators of the dysregulation of various processes in OA pathophysiology^30^. In particular, age-related increase in cytokine levels is the main cause of articular cartilage degeneration in OA. The pro-inflammatory molecules used in this study including lipopolysaccharide (LPS), interleukin 1β (IL-1β), and tumor necrosis factor-α (TNF-α), led to a significant decrease in the viability of chondrogenic cells, except interleukin 6 (IL-6), which is considered a cell proliferation factor in some cases^31–33^ (Fig. 4a). Exposure to LPS markedly inhibited AQP1 mRNA expression (Fig. 4b), whereas AQP3 and AQP4 did not respond to LPS stimulation. TNF-α, a major cytokine in OA physiopathology, specifically reduced AQP3 gene expression (Fig. 4c), but showed no noticeable change in AQP1 and AQP4 expression. Interestingly, the representative cytokines IL-1β and IL-6 had no significant effect on the gene expression of any of the three AQPs, but IL-6 caused an increase in AQP gene expression. Collectively, these results indicate that some pro-inflammatory molecules that are induced by aging may lead to the reduction of chondrocyte AQP gene expression.

### High mobility group box 1 (HMGB1) reduces AQP4 expression in chondrocytes

**Figure 5:**
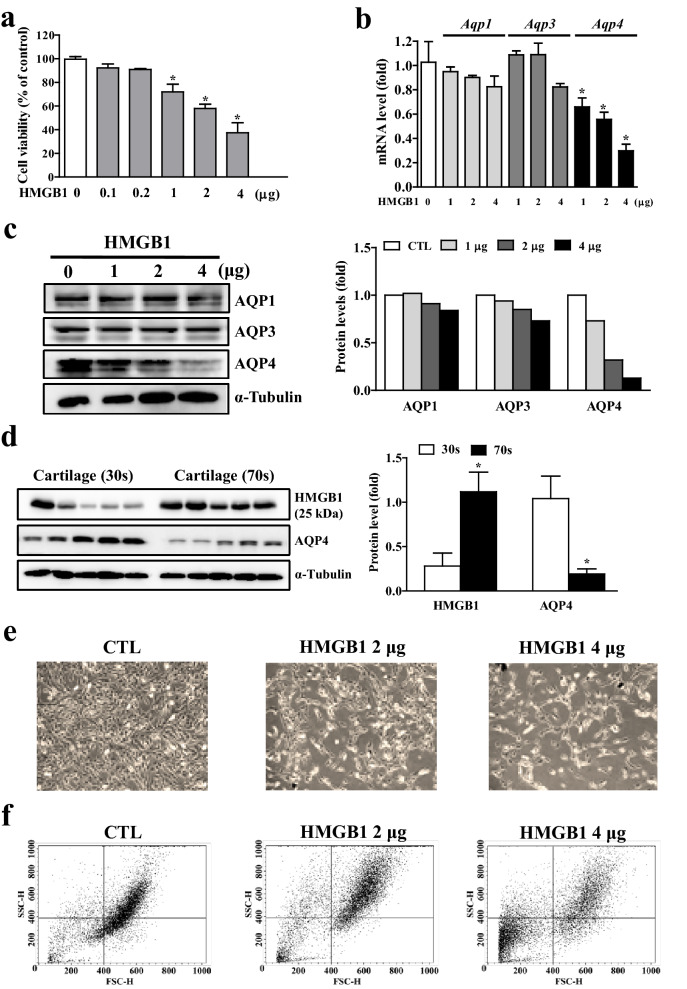
Reduction in aquaporin expression and cell damage by HMGB1. (**a**) Cell viability of chondrogenic C3H10T1/2 cells by HMGB1. (**b**) Expressions of AQP1, -3 and -4 mRNA by HMGB1. (**c**) Western blot analyses for AQP1, -3 and -4 protein expression by HMGB1. Densitometric analyses of the western blots are shown on the right. (**d**) HMGB1 and AQP4 protein expression in young and old aged cartilage tissues (n = 5 per group). Densitometric analyses of the western blots are shown on the right. (**e**) Morphology of chondrogenic C3H10T1/2 cells by HMGB1 administration. (**f**) Flow cytometry analysis in HMGB1-treated C3H10T1/2 cells. Forward scatter (FSC) represent the cell size, and side scatter (SSC) data represent the complexity or granularity of the cell.

Pro-inflammatory molecules, LPS and TNF-α, showed a detrimental effect on AQP1 and AQP3 gene expression, respectively. However, chondrocyte-AQP4 expression was not affected by these molecules, suggesting that neither cytokines nor LPS are responsible for the negative regulation of AQP4 (Fig. 4d). Therefore, we sought to identify the molecule that reduces AQP4 expression in chondrocytes during age-associated degeneration of articular cartilage. We chose to focus on HMGB1 as it is well-known to be associated with immune response^34^, and recent emerging evidence suggests that HMGB1 functions as an important mediator of cellular senescence^35–37^. As shown in Fig. 5a, the viability of chondrogenic C3H10T1/2 cells was significantly decreased following HMGB1 administration. Interestingly, HMGB1 specifically reduced AQP4 gene expression, but had no significant effect on either AQP1 or AQP3 mRNA expression (Fig. 5b). Consistent with the RT-qPCR results, western blot analysis revealed that HMGB1 treatment markedly reduced AQP4 protein level in a dose-dependent manner (Fig. 5c). To assess whether both HMGB1 and AQP4 expression are directly affected by aging, we compared the protein levels in knee articular cartilage between relatively young (30 s) and older (70 s) groups of patients. As shown in Fig. 5d, HMGB1 protein level was significantly increased in the older group, whereas AQP4 protein level was markedly decreased. To examine whether upregulation of HMGB1 leads to any negative effect on chondrocytes, differentiated C3H10T1/2 cells were challenged with HMGB1. As shown in Fig. 5e, HMGB1 caused noticeable cellular damage in a dose-dependent manner, resulting in decreased cell population compared to the control. Furthermore, flow cytometry analysis showed a reduction in the forward scatter (FSC) due to cell shrinkage and an increase in side scatter (SSC), probably due to chromatin condensation (Fig. 5f). Taken together, these results suggest that HMGB1 is elevated in the knee articular cartilage of aged-OA patients and causes a reduction in chondrocyte-AQP4 expression as well as chondrocyte damage.

### HMGB1 neutralization improves HMGB1-induced AQP4 reduction and chondrocyte defects

**Figure 6:**
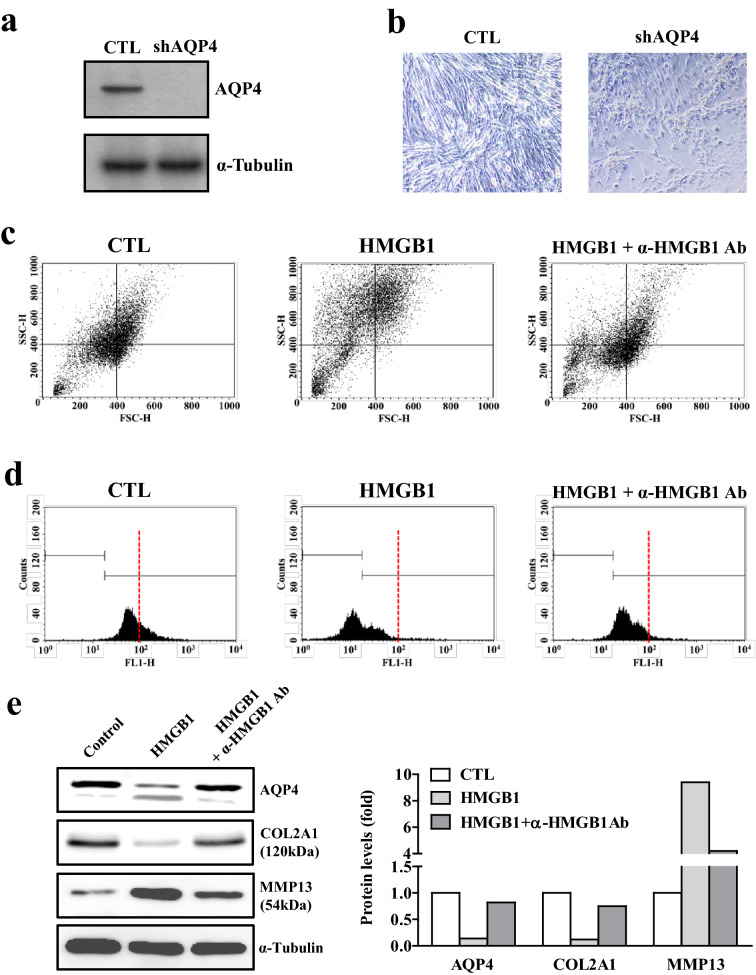
Chondrocyte defects by loss of AQP4 and improvement of HMGB1-induced cell defects by HMGB1 neutralization. (**a**) AQP4 protein expression in AQP4 shRNA-transfected C3H10T1/2 cells. (**b**) Morphology of AQP4 shRNA-transfected chondrogenic C3H10T1/2 cells for 48 h. (**c**) Flow cytometry analysis in HMGB1-treated chondrogenic C3H10T1/2 cells in the presence or absence of HMGB1- antibody (Ab). (**d**) Representative frequency histogram of DiOC6 fluorescence (FL1-H) the showing the mitochondrial membrane potential. (**e**) Western blot analyses for AQP4, COL2A1 and MMP13 protein expression. Densitometric analyses of the western blots are shown on the right.

To verify whether a functional defect in AQP4 substantially correlates with chondrocyte damage, we knocked down AQP4 by transfecting shRNA against AQP4 in C3H10T1/2 cells (Fig. 6a). In the presence of AQP4 shRNA, the chondrogenic C3H10T1/2 cells significantly shrank and the cell population was decreased remarkably compared with control (Fig. 6b), suggesting that AQP4 loss is associated with substantial chondrocyte damage. To check whether HMGB1-mediated chondrocyte damage and AQP4 reduction can be restored by blocking HMGB1, we treated differentiated C3H10T1/2 cells with HMGB1 in the presence or absence of HMGB1-antibodies. As shown in Fig. 6c, HMGB1-induced chondrocyte damage was substantially improved by neutralizing HMGB1. To assess HMGB1-dependent cellular senescence and apoptosis of chondrocytes indirectly, we examined mitochondrial membrane potential by staining with the cationic dye, DiOC6. As shown in Fig. 6d, HMGB1 administration led to a marked depletion of mitochondrial membrane potential, reflecting the initiation of apoptotic signaling. Interestingly, neutralization of HMGB1 led to significant improvement in the mitochondrial membrane potential, suggesting that blockade of HMGB1 may be a potent therapeutic approach for HMGB1-induced chondrocyte damage. With respect to the effect of HMGB1 on chondrocyte function, we observed changes in the chondrogenic genes, as well as AQP4. As shown in Fig. 6e, HMGB1 significantly decreased the protein levels of AQP4 and COL2A1, an anabolic marker for cartilage, whereas it markedly increased the expression of the catabolic marker, matrix metalloproteinase 13 (MMP13). Corroborating the ameliorative effect of HMGB1 blockade on chondrocyte viability, the protein expression of AQP4 and COL2A1 were restored, whereas that of MMP13 was reduced. Taken together, these results suggest that HMGB1 reduces AQP4 expression and induces chondrocyte damage, and these defects can be restored by blocking HMGB1.

## Discussion

Given that the knee articular cartilage is in an aqueous environment, we hypothesized that AQP, which functions as a water transport channel in various types of cells, may play an active role in the articular cartilage, and that its expression may be affected by aging. In the present study, using human knee articular cartilage tissue obtained from patients with arthroscopy surgery or total knee arthroplasty, we observed significant reduction in the expression of AQP1, -3, and -4 mRNA and protein in damaged fibrocartilage and in cartilage tissues from older adults, respectively. In addition, the expression of AQP1, -3, and -4 were progressively regulated during chondrocyte differentiation, together with other chondrogenic genes such as SOX9, COL2A1, and ACAN. In a previous report, AQP1 and AQP3 were shown to be expressed by chondrocytes in articular cartilage, suggesting that AQP1 and AQP3 may play a role in the transport of water or small solutes and osmotically active metabolites across the chondrocyte plasma membrane during volume regulatory behavior^22^. Graziano et al. also reported that the expression of AQP1 and AQP3 were markedly increased during the chondrogenic differentiation of human mesenchymal stem cells derived from adipose tissue, and that the inhibition of AQP1 and AQP3 expression caused a significant decrease in chondrocyte viability and mRNA expression of chondrogenic markers, respectively^24^. These results, together with our similar findings, suggest that expression of AQPs, at least that of AQP1, -3, and -4 are regulated during the chondrocyte differentiation process and may play key roles in a variety of cellular functions such as maintenance of cartilage structure, regulation of fluid flow-induced loading forces, exchange of nutrients or metabolites, and remodeling of the extracellular matrix within articular cartilage an avascular tissue. In contrast, several studies have demonstrated the function of AQP in the cartilage. Haneda et al. reported that AQP1 mRNA expression was significantly higher in OA cartilage, and that IL‑1β treatment increased AQP1 expression in hip explant cartilage^25^. In addition, Cai et al. reported that AQP4 protein expression is increased in cartilage tissues of adjuvant-induced arthritis rat models, and AQP4 inhibition by acetazolamide treatment increased cell proliferation and mRNA levels of COL2A1 and ACAN^26^. With respect to this contradiction, we cautiously speculate that acute increase of AQP expression in OA condition may reflect a compensatory phenomenon to restore AQP function against cartilage damage, which leads to AQP degeneration. Otherwise, it is possible that certain changes in chondrocyte morphology or volume by cartilage water content or synovial fluid osmolality in OA condition may effect on the expression and function of AQPs^38^. A previous study suggesting that AQP expression and function may differ in OA chondrocytes is also worth consideration^39^. Given its intrinsic function, the role of AQPs in water transport in the cartilage seems to be greater than its role as a transcriptional regulator, although the possibility of indirect regulation of gene transcription by AQPs in certain circumstances cannot be ruled out. To better understand the essential role of AQPs in chondrocytes or articular cartilage, a chondrocyte-specific gain or loss of function strategy may be needed.

In the present study, we found that the expression of different types of AQPs were downregulated by different types of age-associated factors. As shown in Figs. 3, 4, 5, mRNA, and protein levels of AQP1, -3 and -4 were significantly reduced by H_2_O_2_, TNFα, and HMGB1, respectively. A previous report demonstrated that H_2_O_2_ decreased the expression level of AQP1, while AQP1 overexpression decreased high glucose-induced mitochondrial ROS generation in bovine aortic endothelial cells^40^. Additionally, it was reported that the expression level of AQP1 in the pulmonary capillary endothelium of mice was decreased by LPS, an inducer of ROS^41^. Regarding AQP3, TNFα decreases AQP3 mRNA expression in intestinal epithelial cells^42^. A recent study demonstrated HMGB1-dependent loss of AQP4 protein expression in atrophied muscle^43^, and identified the molecular mechanism by which muscle injury-induced HMGB1 activation leads to the expression of the representative E3 ligase, atrogin-1, which drives AQP4 degradation via ubiquitination. Collectively, these results indicate that each AQP may have its own susceptibility to an individual or a specific age-associated factor, even if the cell types are different.

HMGB1 is a chromatin protein that exerts divergent effects on cells. HMGB1 participates in the organization of DNA and nucleosomes and regulates gene transcription in the nucleus. During cell injury that causes cell membrane rupture or cell death, nuclear HMGB1 rapidly leaks into the cytoplasm, where it promotes innate and adaptive immune responses and exhibits cytokine activity^44,45^. However, its contribution to immune responses or other regulatory functions in articular cartilage is not fully understood. Herein, we found that aging-induced HMGB1 leads to reduction in AQP4 and chondrocyte defects (Fig. 5). We also evaluated the effect of HMGB1 inhibition using a neutralizing antibody to revoke its detrimental effect on cartilage (Fig. 6c–e). A recent study reported that HMGB1 expression was significantly increased in mouse OA joints, and intra-articular injection of anti-HMGB1 antibodies had cartilage-protective effects that were comparable to treatment with a TNF-α inhibitor^46^. They suggested that HMGB1 affects chondrocyte function by inducing the protein expression of IL6 and IL8 and downregulating mRNA of COL2A1. These results indicate that HMGB1 may be a new target for OA therapy, and clinical application of HMGB1 inhibitors may be a promising therapeutic approach for cartilage degeneration, although the regulatory mechanism of HMGB1 relies on different targets.

The present study has a few limitations that require consideration. First, the sample size was small, which weakened the power of this pilot study. To assess the effect of aging on the expression of AQPs, the samples were divided into three different age groups, which limited the total number of samples. However, we are confident that the data in this pilot study provide meaningful differences of statistical significance given that the ‘n’ was > 12 in each experimental group. In addition, the definitions of ‘hyaline cartilage (used to indicate a normal cartilage)’ and ‘fibrocartilage (used to indicate an injured cartilage)’ in this study are a little dubious. As it was difficult to obtain pure normal knee articular cartilage in this study, we acquired relatively healthy cartilage tissue far from the damaged area to use as hyaline cartilage. However, we are confident that the cartilage tissues used are appropriately defined by the sufficiently skilled and experienced doctors at our institution. With respect to the condition of cartilage tissues obtained from the patients, we also believe that the collected cartilage tissues are handled appropriately, and that the quality of each tissue as cartilage debris and dissected cartilage tissues is similar. The purity of extracted nucleic acid from same amount of each cartilage tissue was quite similar based on the ratio of optical density which was analyzed by a spectrophotometer. In addition, normal saline used for irrigation may not effect on the cartilage tissue degradation. Second, we did not elucidate the molecular mechanisms underlying AQP degeneration caused by each age-associated factor. As a preliminary study, we evaluated the possible role of AQPs in chondrocytes and alterations in their expression caused by various age-induced factors. An in-depth study of the potent molecular mechanisms and identity of critical molecules or signal pathways that mediate AQP degeneration during aging, will need to be conducted in the future. Third, we did not investigate the effects of other inhibitors against age-associated factors on AQP and cartilage degeneration, except for HMGB1 inhibition. Due to the limited scope of the study, we evaluated only the inhibitory effect of HMGB1 as a novel age-associated factor in knee articular cartilage. Further follow-up studies are expected to reveal the possibility of improving AQP or cartilage degeneration by inhibiting these age-associated factors.

In conclusion, our present study demonstrated that the expression of water channel proteins AQP1, -3 and -4 are regulated by chondrocytes during the differentiation process, and their expression is deregulated by specific age-associated factors in the knee articular cartilage. We also elucidated that age-induced HMGB1 is an AQP4-specific negative regulator, and its inhibition improves AQP4 degeneration and cartilage damage. Our findings contribute significantly to a better understanding of the pathways that mediate aging-induced cartilage degeneration. In addition, the clinical application of HMGB1 inhibitors as well as other potent inhibitors against the identified age-associated factors represent a promising therapeutic approach to suppress cartilage degeneration caused by aging.

## Methods

This study was approved by the Public Institutional Review Board designated by the Ministry of Health and Welfare (IRB No. P01-202012-31-002), and written informed consent was obtained from all the patients who agreed to participate in this study. We complied with all relevant ethical regulations for work with human participants, in accordance with the National Institutes of Health Guidelines.

### Subjects and tissue acquisition

Among the patients who were surgically treated for osteoarthritis of the knee by arthroscopic or total knee replacement surgery, we selected 40 patients who had no steroid injection within 3 months prior to the surgery and had no previous history of surgery on the knees. Knee osteoarthritis was classified by the Kellgren-Lawrence (KL) grading scale, the most widely used classification for knee OA, according to Rosenberg view standing anteroposterior (AP) and 45° posteroanterior (PA) flexion weight-bearing radiograph. Patients determined to have KL grade 1, 2 (n = 19) underwent arthroscopic surgery, and those who were determined to have KL grade 3, 4 (n = 21) underwent TKR surgery. The patients were divided into three different age groups as follows: young (mean age, 35.3 ± 5.0 years; eight men and four women), middle (mean age, 58.4 ± 6.2 years; six men and eight women) and old (mean age, 81.1 ± 4.5 years; four men and ten women). Cartilage tissues were obtained by drainage of saline irrigation fluid containing cartilage debris during arthroscopic surgery or by direct dissection of cartilage tissues that covered the surface of a bone using a surgical blade following total knee replacement surgery. The cartilage tissue samples were immediately frozen at  − 80 °C for polymerase chain reaction (PCR) and western blot analyses.

###  Reverse-transcription quantitative PCR (RT-qPCR) analysis

Total RNA was extracted from the isolated cartilage tissues or cultured cells using TRIzol reagent (Invitrogen, Carlsbad, CA, USA) and used for cDNA synthesis. qPCR was carried out on a Light Cycler 480 System (Roche Diagnostics, Switzerland) using 2 × qPCRBIO SyGreen Mix Lo-ROX (PCR Biosystems, London, UK). All data were normalized to β-actin expression and the data were quantitatively analyzed. The primer sequences used are listed in Table 1. All experiments were repeated at least three times.

**Table 1:** Primers used for quantitative RT-PCR.

Gene full name	Gene symbol	Sequences; forward (F) / reverse (R)
(Human) Aquaporin 1	AQP1	(F) 5'-CTGGGCATCGAGATCATCGG-3' (R) 5'-ATCCCACAGCCAGTGTAGTCA-3'
(Human) Aquaporin 3	AQP3	(F) 5'-GGGGAGATGCTCCACATCC-3' (R) 5'-AAAGGCCAGGTTGATGGTGAG-3'
(Human) Aquaporin 4	AQP4	(F) 5'-AGCAGTCACAGCGGAATTTCT-3' (R) 5'-TCTGTTCCACCCCAGTTGATG-3'
(Mouse) Aquaporin 1	Aqp1	(F) 5'-AACCACTGGATTTTCTGG-3' (R) 5'-CTTCATCTCCACCCTGGA-3'
(Mouse) Aquaporin 3	Aqp3	(F) 5'-CACATCCGCTACCGGCTG-3' (R) 5'-TAGATGGGCAGCTTGATC-3'
(Mouse) Aquaporin 4	Aqp4	(F) 5'-TTGCTTTGGACTCAGCATTG-3' (R) 5'-AACCAGGAGACCATGACCAG-3'
(Mouse) SRY-box containing gene 9	Sox9	(F) 5'-AGTACCCGCATCTGCACAAC-3' (R) 5'-ACGAAGGGTCTCTTCTCGCT-3'
(Mouse) Collagen, type II, alpha 1	Col2a1	(F) 5'-GGGTCACAGAGGTTACCCAG-3' (R) 5'-ACCAGGGGAACCACTCTCAC-3'
(Mouse) Aggrecan	Acan	(F) 5'-GTGGAGCCGTGTTTCCAAG-3' (R) 5'-AGATGCTGTTGACTCGAACCT-3'
(Mouse) Glyceraldehyde-3-phosphate dehydrogenase	Gapdh	(F) 5'-ATAATACCGATCCCCGAAGG-3' (R) 5'-CTGGATGGTGTATGCACAGG-3'
(Human/mouse) Actin, beta	Actb	(F) 5'-TCTGGCACCACACCTTCTAC-3' (R) 5'-TCGTAGATGGGCACAGTGTGG-3'

### Western blot analysis

Cells were lysed with radioimmunoprecipitation assay (RIPA) lysis buffer, separated by 10% sodium dodecyl sulfate polyacrylamide gel electrophoresis (SDS-PAGE), and transferred to a polyvinylidene fluoride (PVDF) membrane. To observe the protein expression simultaneously and to ensure the same experimental conditions instead of membrane duplication, we cut the nitrocellulose membrane after transfer into 2 ~ 3 parts by the size; 25 ~ 48 kDa for AQP1 (28 kDa), AQP3 (31 kDa) and AQP4 (34 kDa), 48 ~ 75 kDa for MMP13 (54 kDa) or α-tubulin (55 kDa), 75 ~ 245 kDa for COL2A1 (120 kDa). The membranes were probed with anti-AQP1, -3, -4 (sc-25287, sc-9885, sc-390488, Santa Cruz Biotechnology, Dallas, TX, USA), anti-HMGB1 (ab79823, Abcam, Cambridge, MA, USA), anti-COL2A1 (clone 6B3, MAB8887, Sigma-Aldrich, St. Louis, MO, USA), anti-MMP13 (ab39012, Abcam), and anti-α-tubulin (ab7291, Abcam) antibodies. Immunoreactive proteins were assessed using an LAS-3000 image analyzer (Fuji Film, Tokyo, Japan). The original blot images are provided in ‘Supplementary Figures'. The proteins were quantified by densitometry using ImageJ software (Ver.1.8.0; https://imagej.nih.gov/ij, National Institutes of Health, Bethesda, MD, USA).

### Cell culture and chondrogenic differentiation

C3H10T1/2 murine mesenchymal stem cells were cultured in Dulbecco’s modified Eagle’s medium (Corning, Corning, NY, USA) supplemented with 10% fetal bovine serum (FBS, Gibco, Thermo Fisher Scientific, USA) and antibiotics at 37 °C in a humidified atmosphere containing 5% CO_2_. To induce chondrogenic differentiation, the cells were treated with differentiation medium containing 100 ng of bone morphogenetic protein 2 (BMP2), as described in a previous report^28^ and the medium was changed every three days and fresh BMP-2 was added.

### Cell viability assay and reagents

Cell viability of chondrogenic C3H10T1/2 cells was assessed using the 3-(4, 5-dimethylthiazol-2-yl)-2, 5-diphenyltetrazolium bromide (MTT, M2128, Sigma-Aldrich, St. Louis, MO, USA) assay. Briefly, chondrogenic C3H10T1/2 cells were plated at a density of 1 × 10^4^ cells/well in 24-well culture plates and incubated for 24 h at 37 °C and 5% CO_2_. The next day, the culture medium was replaced with fresh Dulbecco’s modified Eagle’s medium (DMEM; vehicle control) or DMEM containing different concentrations of reagents: H_2_O_2_ (Sigma-Aldrich), LPS (E. coli 026:B6, L2654, Sigma-Aldrich), IL-1β (Cell Signaling Technology, Danvers, MA, USA), IL-6 (CYT-213, PROSPEC, Israel), TNFα (Cell Signaling Technology), and HMGB1 (BioLegend Inc., San Diego, CA, USA). At 48 h after reagent treatment, MTT (5 mg/mL) was added, and the culture plates were incubated at 37 °C for 4 h. The resulting formazan product was dissolved in DMSO, and the absorbance was measured at 560 nm using an Ultra Multifunctional Microplate Reader (Tecan US, Inc., Morrisville, NC, USA). Cell viability was determined from the readings using the formula % Viability = (fluorescence value of MSM/fluorescence value of non-treated control) × 100. All measurements were performed in triplicate, and the experiments were repeated at least thrice.

### Flow cytometric analysis

Chondrogenic C3H10T1/2 cells were plated at a density of 1 × 10^5^ cells/well in 6-well plates and incubated for 24 h. The cells were then treated with different doses of HMGB1 in DMEM for an additional 48 h. The harvested cells were washed twice with PBS and suspended in PBS (0.5 mL). The size and granularity of cells were determined by forward and side scatter (FSC and SSC) gating, respectively, using flow cytometry on a FACScalibur instrument and analyzed with CellQuest software (Ver. 3.3; https://www.bd.com, BD Bioscience, San Jose, CA, USA). The uptake of 3,3'-dihexyloxacarbocyanine iodide (DiOC6(3)) was analyzed to measure the loss of mitochondrial membrane potential, reflecting the initiation of the pro-apoptotic signal.

### Knockdown of AQP4

For loss-of-function experiments, lentiviral particles for AQP4 shRNA were generated and transfected in chondrogenic C3H10T1/2 cells. shRNA specific to the mouse AQP4 gene and the control construct, pLKO-GFP, were commercially purchased (AQP4 MISSION shRNA, SHCLNG-NM_009700, Sigma-Aldric). The sequence of the shRNA targeting the AQP4 mRNA was 5’-CCGGCAATTGGACATTTGTTTGCAACTCGAGTTGCAAACAAATGTCCAATTGTTTTTG-3’.

### Statistical analyses

Data are expressed as mean ± standard error. Differences between the control and experimental groups were assessed using t-tests (α = 0.05) in GraphPad Prism 5.01 (GraphPad Software, Ver. 5.01; https://www.graphpad.com, La Jolla, CA, USA). Differences were considered significant when P values were < 0.05.

## Supplementary Information

Supplementary Figures.
