# Supplementary material for: Differential regulation of the water channel protein aquaporins in chondrocytes of human knee articular cartilage by aging
Source: Sci Rep. 2021 Oct 14;11:20425. doi: 10.1038/s41598-021-99885-7 (PMC8516946; doi:10.1038/s41598-021-99885-7)
Supplement: Supplementary file 1 — Supplementary Figures. [file 41598_2021_99885_MOESM1_ESM.pdf]

Figure 1

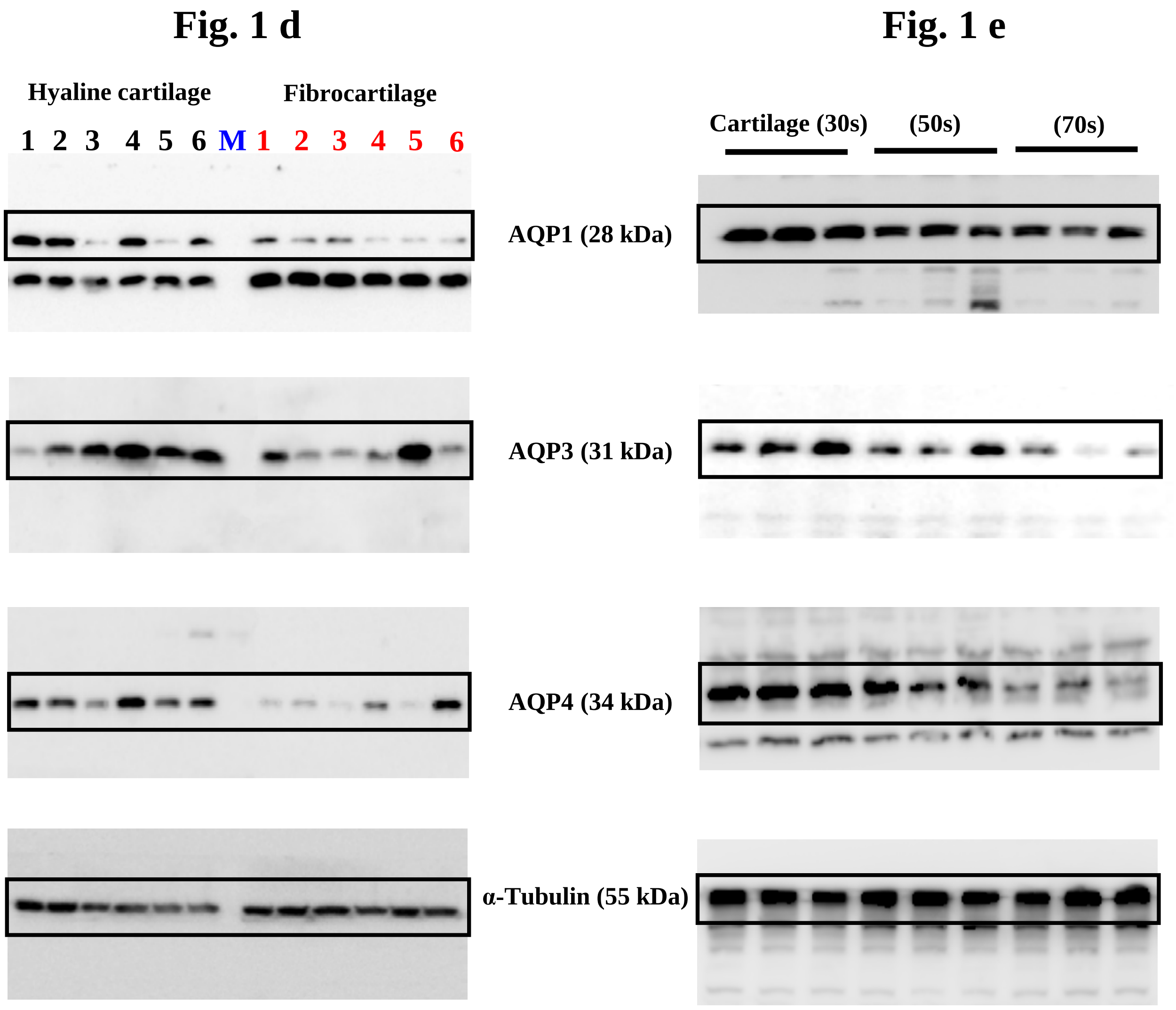
Fig. 1 d
Hyaline cartilage
Fibrocartilage
1 2 3 4 5 6 M 1 2 3 4 5 6
Fig. 1 e
Cartilage (30s)
(50s)
(70s)
AQP1 (28 kDa)
AQP3 (31 kDa)
AQP4 (34 kDa)
α-Tubulin (55 kDa)

Figure 2

Fig. 2 b

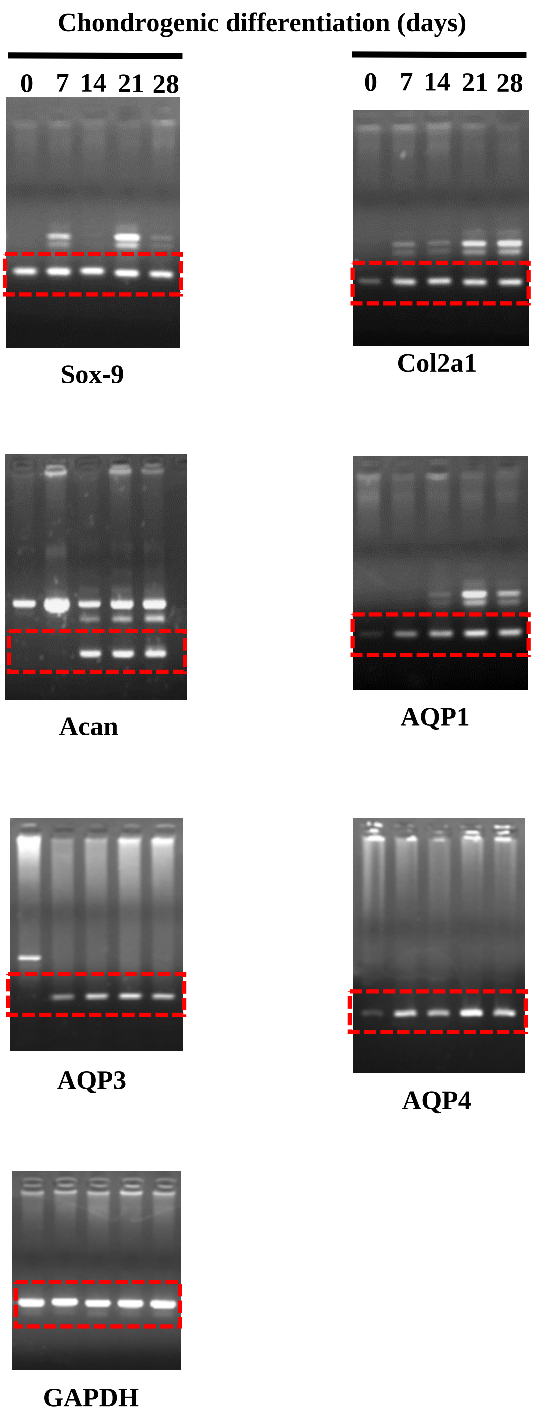

Fig. 2 c

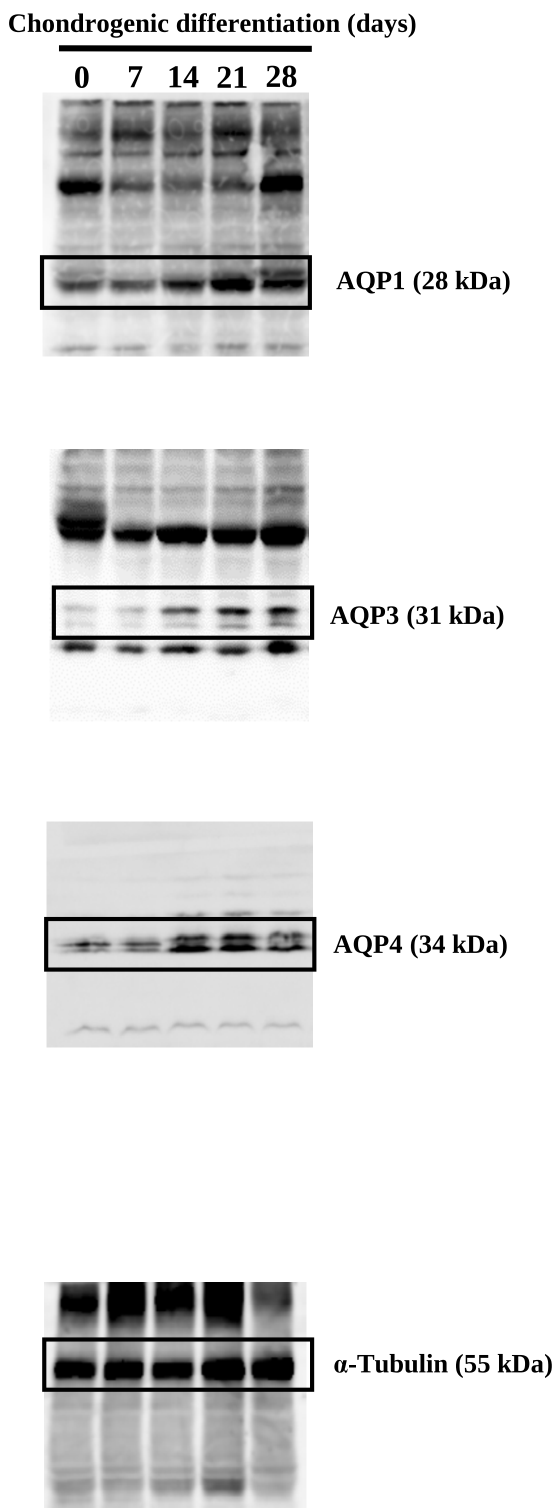

Figure 3

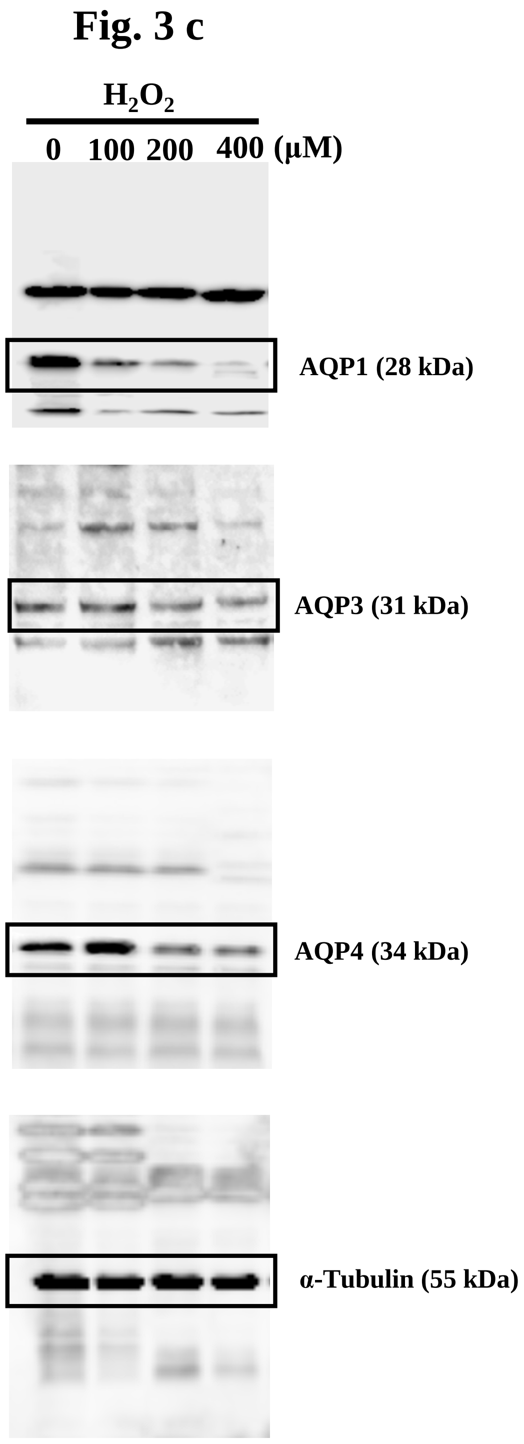

Fig. 3 c
H_2O_2
0 100 200 400 (µM)
AQP1 (28 kDa)
AQP3 (31 kDa)
AQP4 (34 kDa)
α-Tubulin (55 kDa)

Figure 5

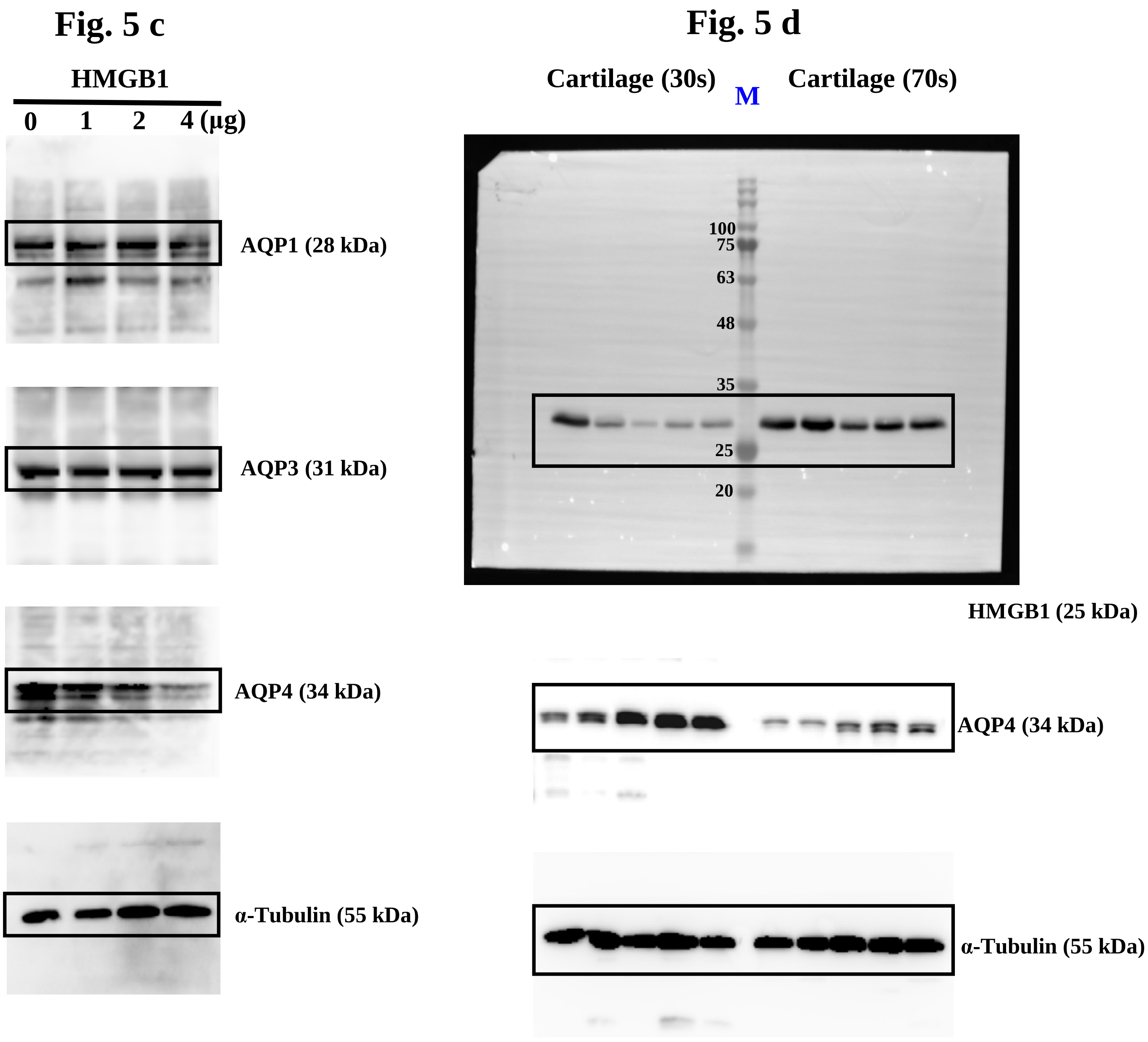

Fig. 5 c
HMGB1
0 1 2 4 (µg)
AQP1 (28 kDa)
AQP3 (31 kDa)
AQP4 (34 kDa)
α-Tubulin (55 kDa)
Fig. 5 d
Cartilage (30s)
M
Cartilage (70s)
100
75
63
48
35
25
20
HMGB1 (25 kDa)
AQP4 (34 kDa)
α-Tubulin (55 kDa)

Figure 6

Fig. 6 a

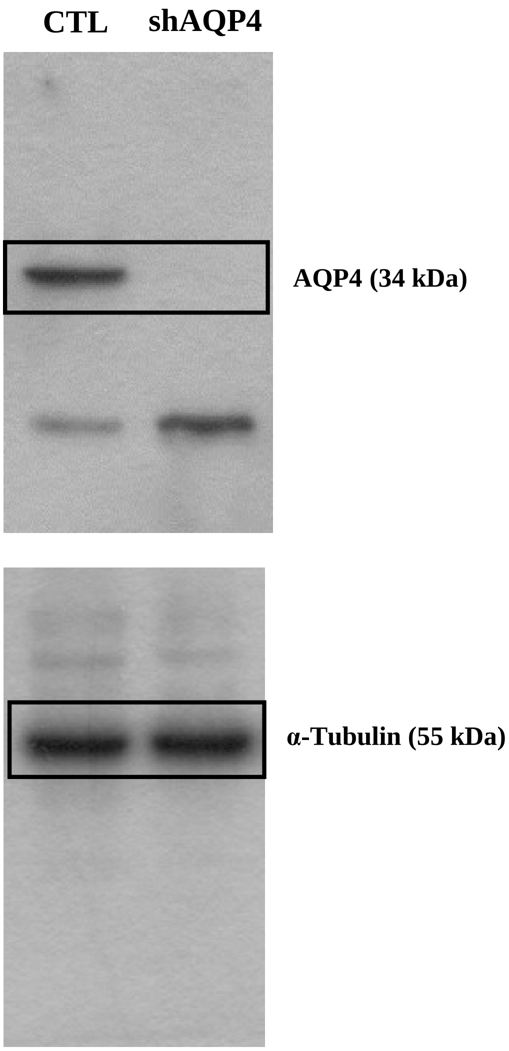

Fig. 6 e

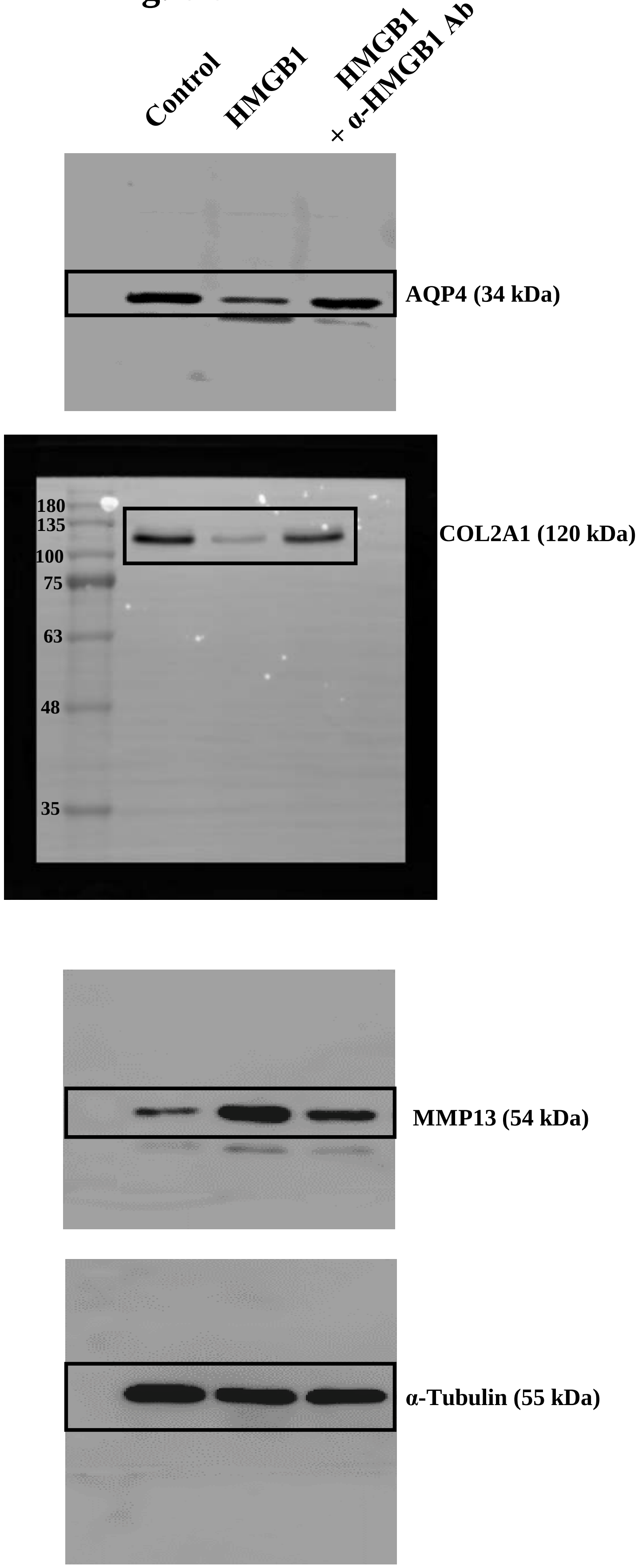

Notice

To observe the protein expression simultaneously and to ensure the same experimental conditions instead of membrane duplication, we cut the nitrocellulose membrane after transfer into 2~3 parts by the size; 25~48 kDa for AQP1 (28 kDa), AQP3 (31 kDa) and AQP4 (34 kDa), 48~75 kDa for MMP13 (54 kDa) or α-tubulin (55 kDa), 75~245 kDa for COL2A1 (120 kDa), and then the membranes were blotted with each antibody.
In addition, when we take the photo using image analyzer, we saved the image with the smallest as much as possible to reduce the data saving volume by cropping the exact band area, even without size marker. All the images in the Supplementary Information file are the original, unprocessed versions of images. However, we humbly admit that there was carelessness in the preparation of data figures, although we confident that the protein bands were the precise signal with corresponding protein size.
